# Orthodontic treatment of children/adolescents with special health care needs: an analysis of treatment length and clinical outcome

**DOI:** 10.1186/1472-6831-14-67

**Published:** 2014-06-11

**Authors:** Moritz Blanck-Lubarsch, Ariane Hohoff, Dirk Wiechmann, Thomas Stamm

**Affiliations:** 1 Department of Orthodontics, University Hospital Muenster, Westphalian Wilhelms-University, Muenster, Germany; 2 Department of Orthodontics, Hannover Medical School, Hannover, Germany

**Keywords:** Orthodontic treatment, Children/adolescents with special health care needs (SHCNs), Treatment time, Success, Peer assessment rating (PAR) index, Index of orthodontic treatment need (IOTN), Aesthetic component (AC)

## Abstract

**Background:**

The aim of this retrospective study was to analyse the treatment time and differences between the pre- and post-treatment peer assessment rating (PAR) index and aesthetic component (AC) of the index of orthodontic treatment need (IOTN) scores in children/adolescents with special health care needs (SHCNs), compared to non-special health care needs (NSHCNs) controls.

**Methods:**

Based on certain inclusion and exclusion criteria, medical records of SHCNs and randomly selected NSHCNs controls at the Department of Orthodontics, University Hospital Muenster were analysed retrospectively for the treatment time, number of appointments, chair time (“moderate” or “considerable”), PAR scores, and AC scores. Sample size calculation, descriptive statistics, and explorative analyses were performed using the Mann–Whitney *U* Test.

**Results:**

Twenty-nine children with SHCNs (21 boys, 9 girls; median age: 11 years, pre-treatment) and 29 children with NSHCNs (12 boys, 17 girls; median age: 12 years, pre-treatment) were enrolled in this study.

The overall treatment time did not differ between the patient groups. However, more “considerable chair time” was needed for the SHCNs group compared to the control group (p < 0.05), whereas “moderate chair time” was more often needed in patients with NSHCNs (p = 0.001).

The age of the patients at the first and last appointments showed significant statistical differences: children in the SHCNs group commenced orthodontic treatment earlier, by a median of 1 year, compared to children in the NSHCNs group.

The SHCNs group had significantly higher pre- and post-treatment PAR scores (median 21/median 6) and AC scores (median 9/median 3) compared to NSHCNs patients (PAR: median 17/median 0; AC: median 5/median 1).

However, the overall treatment time and the overall PAR and AC score reduction did not differ significantly between the SHCNs and NSHCNs groups.

**Conclusions:**

While the overall treatment time and number of appointments did not differ, the overall chair time was higher in the SHCNs group. The pre- and post-treatment PAR and AC scores were significantly higher in the SHCNs group.

## Background

According to the World Health Organisation (WHO) definition, “craniofacial abnormalities or anomalies (CFAs) are congenital structural deformities, malformations, or other abnormalities of the skull (cranium) or facial bones. Most common forms of CFA appear to arise from a combination of genetic factors and environmental influences” [1].

Owing to medical advances and an increasing number of treatment alternatives, fewer gravidity- and delivery-related complications are observed in the developed world, and even early born infants, who may suffer from developmental or environmental restrictions on health, have significantly higher survival chances. Consequently, the number of children with special health care needs (SHCNs) continues to rise [2]. Therefore, the integration of people with special health care needs and their families into the mainstream daily and social life is becoming increasingly important [3,4].

A subgroup of the wide spectrum of challenged people is the group of individuals with CFAs. Although CFAs have varied causes and diverse treatment approaches, there is one feature that all individuals with CFAs have in common: they share a unique facial appearance which, unlike that in internal medical disorders that remain undiscovered by the public, exposes them to society [3,5]. As scientifically proven, facial, and dental appearance make a difference in social integration; thus, penalising this patient collective at an early age. Shaw et al. demonstrated that dentofacial appearance influences social attractiveness. They found that people with normal dental appearance were perceived to be better looking, more desirable to make friends with, more intelligent, and less likely to show aggressive behaviour [6-8]. According to a survey led by Becker et al., the primary motivation for parents to have their children with SHCNs undergo orthodontic therapy was to increase their facial attractiveness [3]. Consequently, this knowledge is reason enough to focus on paving the way for patients with CFAs to access orthodontic treatment. Further, the fact that malocclusion occurs more often in children with SHCNs constitutes an additional important reason for this focus [9-11].

Current literature suggests that treatment of this challenged patient collective is possible, but not easy in management, and comparable data on treatment results are rare [12]. Becker et al. outlined that the major issues associated with orthodontic treatment of children with SHCNs are the difficulties in maintenance of adequate oral hygiene and appointment attendance [13].

As malocclusion and aesthetics are often seen as subjective criteria, only the use of standardised indices is capable of shedding light on this highly distinctive patient collective. A manifold number of indices for orthodontic assessment have been described [14]. Two firmly established indices are the peer assessment rating (PAR) index and the index of orthodontic treatment need (IOTN), which were applied in our study.

The PAR index provides a way to define the individual occlusal non-conformance in relation to the entire malocclusion of the jaw, and to draw comparisons between different patients’ cases at variable points of treatment. To meet these requirements, the index is able to uncover all the potential occlusal anomalies known. Improvements in occlusion, indicating success in treatment, can be evaluated by changes in the score [15,16].

The IOTN consists of a clinical component called the dental health component (DHC) and an aesthetic component (AC) [17]. The index differentiates between 3 treatment categories: “no treatment need” (1–4), “borderline need” (5–7), and “great treatment need” (8–10) [18]. Furthermore, the aesthetic component is able to give suggestions on patient cooperation [18].

Further in our study, a Pubmed search was applied to the term “orthodont*” crossed with a combination of “handicapped OR special need OR disabled” in relation to “success,” “outcome,” and “treatment time.” According to the published literature, a focus has been laid on the challenge of treating patients with SHCNs, however, there is little reference to the orthodontic treatment outcomes in this special needs group [3,4,11-13,19-22]. Consequently, the aim of the study was to analyse treatment time and differences between pre- and post-treatment PAR and AC scores of IOTN, in a patient group with SHCNs compared to a control group with non-special health care needs (NSHCNs).

## Methods

### Subjects

Medical records from 1989 to 2008 of the Department of Orthodontics, University Hospital Muenster were screened for orthodontic treatment of children with SHCNs. The term “SHCNs” was defined according to the International Classification of Functioning, Disability and Health (ICF) [23].

### Inclusion criteria

The inclusion criteria for the SHCNs group were: (1) children/adolescents with craniofacial abnormalities according to the WHO definition, (2) treatment with removable (U-bow activator, functional regulator, or palatal plate according to A. M. Schwarz) and/or multibracket appliances (Ormco Corporation, CA, USA), and (3) photographic and model documentation at the beginning and end of treatment. Written informed consent was obtained either from the participants or their legal guardians for data analysis and publication of the associated images.

### Exclusion criteria

The exclusion criteria were: (1) patients with history of orthodontic treatment, (2) adult patients, (3) incomplete medical records, or (4) discontinuation of treatment.

### Control group

The control group with NSHCNs consisted of randomly selected healthy children/adolescents treated at the Department of Orthodontics with removable (U bow activator, functional regulator or palatal plate according to A. M. Schwarz) and/or multibracket appliances (Ormco Corporation, CA, USA). Inclusion criteria No. 2 and 3, as well as all the exclusion criteria of the SHCNs group were applied to the NSHCNs group.

To detect any variation in the treatment time, the date of treatment admittance and date of orthodontic treatment completion were extracted from the medical records. The date of birth was also identified to compare the age of the patient at the time of the first and last appointments. Furthermore, the medical disorder, gender, type of appliance used (fixed or removable), and number of appointments—itemised on the basis of chair time as either “moderate” or “considerable”—were recorded. As a consequence of the retrospective nature of the study, we defined “moderate chair time” as less time consuming orthodontic treatments such as changing elastics or power chains, or wire bending (removable appliances), whereas “considerable chair time” implied procedures like wire change, bracket bonding or rebonding, dental imprint, or first adjustment of a removable appliance.

In order to examine differences between the collective with SHCNs and the control group, standardised indices were applied. The peer assessment rating (PAR) index was used to examine improvement in occlusion between pre- and post-treatment, and to compare the overall treatment outcome between the 2 groups. To record the PAR index, a qualified examiner scored all pre- and post-treatment dental study models. Thus, the occlusal traits of the 11 components (Upper right segment, Upper anterior segment, Upper left segment, Lower right segment, Lower anterior segment, Lower left segment, Right buccal occlusion, Overjet, Overbite, Centreline, and Left buccal occlusion) of the PAR index were recorded and summed. To conduct the analyses, a PAR ruler was used [15].

Further, the aesthetic component (AC) of the index of orthodontic treatment need (IOTN) was recorded to assess the change in the patient’s dental attractiveness over the entire treatment time [24]. The AC of the IOTN requires the examiner to compare the patient’s frontal intraoral photographs with 10 standardized photographs that range from 1, for the most attractive, to 10, for the least attractive dental arrangement [25].

### Statistics

Analyses were performed using the SPSS Statistics Release 21.0 software (IBM Corporation, NY, USA). Metric variables were described by median and range (minimum, maximum). For categorical variables, absolute frequencies were given. Association between a metric outcome variable and a binary predictor variable was assessed using the Mann–Whitney *U* test.

The following 2 null hypotheses were tested on a local two-sided significance level of 5% using the Mann–Whitney *U* test:

Null hypothesis H1: The overall treatment time (defined as the time interval between the start and end of active treatment) in the SHCNs group does not differ from the overall treatment time in the NSHCNs group.

Null hypothesis H2: The reduction in AC scores (defined as the difference between the pre- and post-treatment AC scores) in the SHCNs group does not differ from the reduction in AC scores in the NSHCNs group.

Sample size calculation:

Null hypothesis H1: Sample size calculation for the Mann–Whitney *U* test was performed according to Noether [26] under the assumption of a normally distributed outcome variable [26]. Assumptions on effect sizes were made in line with the guidelines of the German Health Insurance for active treatment time (36 month) and retention (12 month). Any further treatment had to be requested at the health insurance company, meaning that the costs increased for the case. Therefore, mean active treatment time was estimated to be 36 months for the NSHCNs group and 48 months for the SHCNs group. A standard deviation of 12 months in both groups was defined, corresponding to an effect size (standardized difference of means) of d = 1. Under the assumption of an effect size of d = 1 and an allocation ratio of 1 between both groups, the local power is at least 80% for allocated local two-sided significance level of 5%, if the analysis is performed with at least 20 observations in each group.

Null hypothesis H2: Sample size calculation for the Mann–Whitney *U* test was performed according to Noether [26] under the assumption of a normally distributed outcome variable [26]. Under the assumption of a mean AC score for the group with NSHCNs located at 6 (middle of borderline treatment need) and a mean AC score for the group with SHCNs located at 9 (middle of great treatment need), with a common standard deviation of 3 corresponding to an effect size (standardized difference of means) of d = 1 and an allocation ratio of 1 between both groups, the local power is at least 80% for allocated local two-sided significance level of 5%, if the analysis is performed with at least 20 observations in each group.

All analyses were regarded as explorative and p-values were interpreted descriptively. Therefore, no adjustment for multiple testing was performed and all p-values were regarded as local p-values. The local significance level was 5% and the label “significance” used in this study is to be understood as local significance.

## Results

### Subjects

On the basis of the inclusion criteria, 29 children/adolescents with CFAs—composed of Down’s Syndrome, Apert/Crouzon Syndrome, Goldenhar Syndrome, Cerebral palsy, Nager Syndrome, Cleft lip/palate, Treacher Collins-Franceschetti Syndrome and Gorlin-Goltz Syndrome—were enrolled in this study. The patients’ median age was 11 (range: 3–15) years at the start and 14 (range: 8–20) years at end of treatment. The group comprised of 21 boys and 8 girls. Eighteen of them were treated with both fixed and removable appliances, while 7 were treated with fixed appliances only and 4 by removable appliances only.

### Control group

Consistent with the inclusion criteria, 29 children/adolescents with NSHCNs with a median age of 12 (range: 6–18) years at the start and 16 (range: 6–22) years at end of treatment were enrolled in this study. The group comprised of 12 boys and 17 girls. Twenty-six of them were treated with fixed and removable appliances, while 1 was treated with a fixed appliance only and 2 with removable appliances only.

### Treatment time

**Figure 1:**
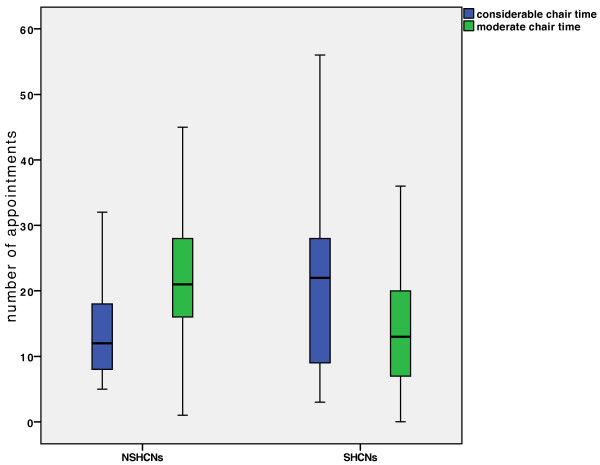
**Number of appointments.** More “considerable chair time” was needed in the group with SHCNs compared to the control group (p < 0.05), whereas “moderate chair time” was more often needed in patients with NSHCNs (p = 0.001).

**Figure 2:**
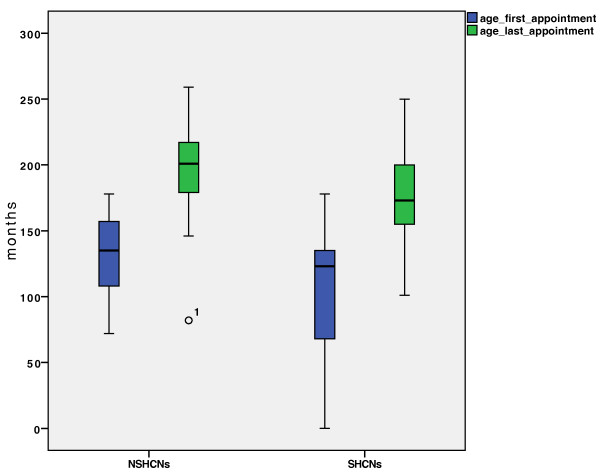
**Age at the time of the first and last appointments.** There is a significant difference between the SHCNs and NSHCNs groups with respect to age at the time of the first (p = 0.018) and last appointments (p = 0.021).

**Figure 3:**
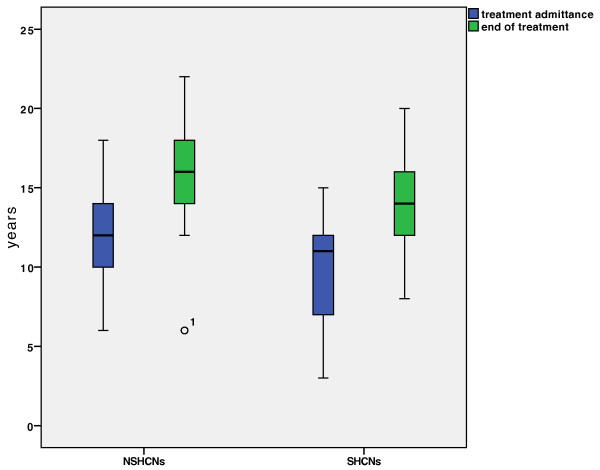
**Age at the time of orthodontic treatment admittance and end of orthodontic treatment.** Children/adolescents with SHCNs commenced orthodontic treatment earlier by a median of 1 year compared to children/adolescents with NSHCNs.

There were no statistically significant differences in the overall treatment time and the number of appointments between the 2 groups.Additionally, the appointments were itemised on the basis of chair time as either “moderate” or “considerable” (Figure 1). This revealed that while more “considerable chair time” was utilized in the SHCNs group compared to the control NSHCNs group (p < 0.05), “moderate chair time” was more often needed in the NSHCNs group (p = 0.001).The age of the patient at the time of the first and last appointments showed significant differences (Figure 2). While the SHCNs group started treatment at a median age of 11 (range: 3–15) years and completed treatment at a median age of 14 (range: 8–20) years, the NSHCNs group started treatment at a median age of 12 (range: 6–18) years and were at a median age of 16 (range: 6–22) years at the end of treatment. Thus, the SHCNs group commenced orthodontic treatment earlier by a median of 1 year compared to the NSHCNs group (Figure 3).

### PAR index

**Figure 4:**
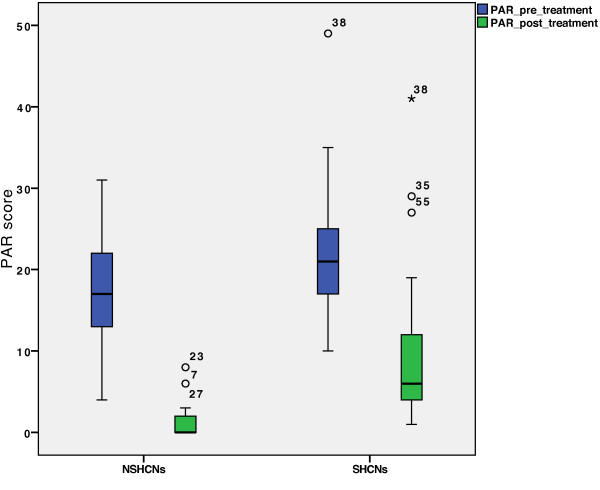
**Pre- and post-treatment PAR scores of the SHCNs and NSHCNs groups.** There are significant differences in PAR scores within the groups, pre-treatment (p = 0.037) and post-treatment (p < 0.001).

**Figure 5:**
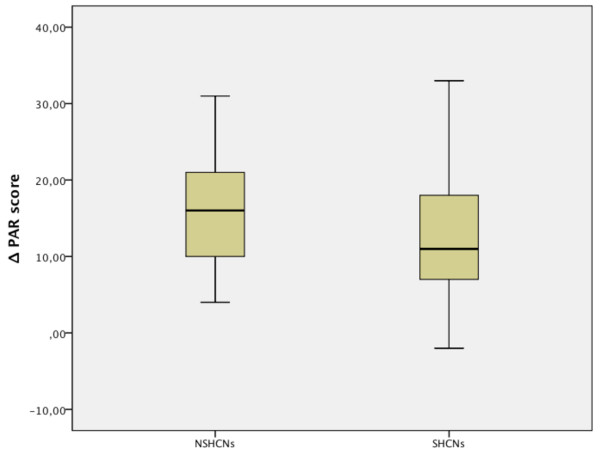
**Reduction in the PAR scores.** ΔPAR (defined as difference between pre- and post-treatment PAR scores).

While a PAR score of 0 indicates perfect alignment and occlusion, higher scores (rarely beyond 50) indicate increasing levels of irregularity. The PAR index was applied to both the start and end of treatment dental study models. The change in the total score reflects the success of the treatment in achieving overall alignment and occlusion [14]. The PAR scores at treatment admittance varied significantly between the control group and the SHCNs group. The PAR score at the end of treatment varied significantly between the SHCNs group (PAR median value: 6) and the NSHCNs group (PAR median value: 0). Further, there was a difference in the boxplot, showing a wider range for the NSHCNs group (Figure 4).With respect to the PAR score reduction (Figure 5), no statistically significant differences could be found between the groups. The score reduction had a median value of 11 (range: -2–33) in the SHCNs group, and 16 (range: 4–31) in the NSHCNs group.

### Aesthetic component

**Figure 6:**
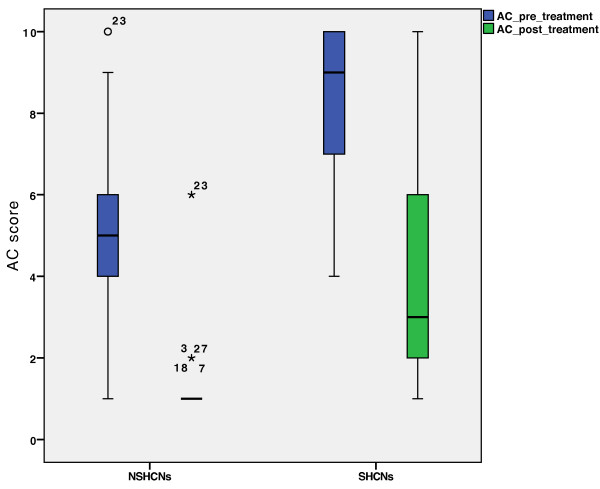
**Pre- and post-treatment AC scores of the SHCNs and NSHCNs groups.** There are significant differences in AC scores within the groups, pre-treatment (p < 0.001) and post-treatment (p < 0.001).

The AC consists of a 10-grade scale illustrated by numbered, colour intraoral photographs. The photographs represent 3 treatment categories: “no treatment need” (grades 1–4), “borderline need” (grades 5–7), and “great treatment need” (grades 8–10) [18]. The AC showed significant differences at treatment admittance between the SHCNs and NSHCNs groups (p < 0.001). The AC’s interquartile range for the SHCNs group was from 7 to 10, while the control group showed an interquartile range of 4 to 6 (Figure 6). Correspondingly, as defined by the AC score, the SHCNs group had a “great treatment need,” while the NSHCNs group had a “borderline treatment need.” The AC at treatment completion also varied significantly between the SHCNs and NSHCNs groups (p < 0.001).

**Figure 7:**
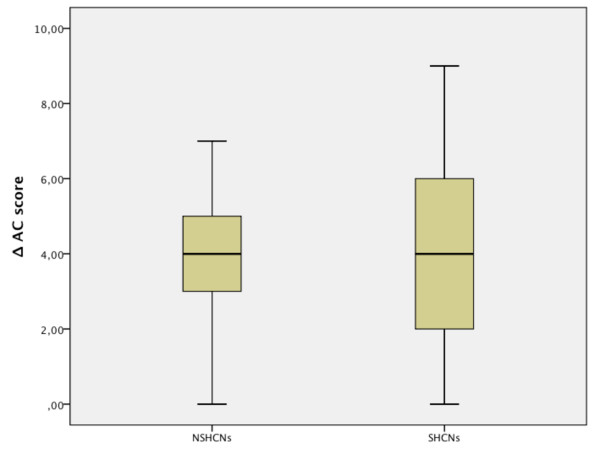
**Reduction in the AC scores.** ΔAC (defined as difference between pre- and post-treatment AC scores).

However, the reduction in the AC score between pre- and post-treatment was the same for both groups (median value: 4) and did not reach statistical significance (Figure 7) (Table 1).

**Table 1:** **Descriptive statistics of the entire data collected, including the total number of the patient collective, division into children/adolescents with and without SHCNs, and the p value of the Mann–Whitney *U *** **test**

	**Total N/median/min/max**	**SHCNs group N/median/min/max**	**NSHCNs group N/median/min/max**	p*
PAR pre treatment	58/19/4/49	29/21/10/49	29/17/4/31	0.037
PAR post treatment	58/2/0/41	29/6/1/41	29/0/0/8	0.000
PAR score reduction	58/13.5/-2/33	29/11/-2/33	29/16/4/31	0.053
AC pre treatment	58/7/1/10	29/9/4/10	29/5/1/10	0.000
AC post treatment	58/2/1/10	29/3/1/10	29/1/1/6	0.000
AC score reduction	58/4/0/9	29/4/0/9	29/4/0/7	0.838
Overall treatment time	58/48/4/186	29/50/4/186	29/48/6/126	0.451
Number of appointments	58/32.5/5/73	29/36/5/70	29/31/6/73	0.652
Number of moderate chair time appointments	58/17/0/45	29/13/0/36	29/21/1/45	0.001
Number of considerable chair time appointments	58/15.5/3/56	29/22/3/56	29/12/5/32	0.048
Age at treatment start	58/11/3/18	29/11/3/15	29/12/6/18	0.028
Age at treatment end	58/15/6/22	29/14/8/20	29/16/6/22	0.025

*p-value of the Mann–Whitney *U* test (comparison of children/adolescents with and without SHCNs).

## Discussion

There are very few studies discussing the orthodontic treatment of children with SHCNs. The current literature focuses on the problems with treatment and lack of care [4,13,27] for this patient collective. The aim of this study was to identify if there are any differences, in terms of treatment length and clinical outcome, between patients with SHCNs and a NSHCNs control group.

### Strengths and limitations of the study

The WHO definition for patients with CFAs leads to a heterogeneous subject group. However, it is precisely this group that is more often affected by malocclusion, therefore this heterogeneous group represents patients with SHCNs in daily practice [9-12]. The indices applied are well established, standardized, and scientifically examined [15,28]. As shown by Brown and Ingelhart, there has to be a reason why orthodontists hesitate to provide care for patients with SHCNs. Hence, a focus was laid on detecting differences between the groups that may influence daily practice and increase the cost of treating patients with SHCNs. As for the statistical analysis, the effect size of the sample size calculation corresponds to the findings mentioned above, even though it may appear to have a wide range [27].

### Treatment time

As demonstrated by our results, the overall treatment time in SHCNs and NSHCNs groups is equal and does not differ in a statistically significant way. The same holds true for the total number of appointments.

With regard to the post-treatment outcome, the question is raised if SHCNs children/adolescents are in need of longer orthodontic treatment to achieve results similar to those of the group with NSHCNs. However, Becker et al. identified the absence of an adequate level of oral hygiene leading to the premature termination of orthodontic treatment as the major reason for insufficient success [13].

Taking a closer look at the differentiation between “considerable-” and “moderate chair time”, there is a difference between the SHCNs and NSHCNs groups. Children/adolescents with SHCNs showed a higher rate of utilization of “considerable chair time,” while children/adolescents with NSHCNs showed a higher rate of utilization of “moderate chair time.” This could be explained by the mental and physical limitations of the SHCNs patients, which can lead to more complications with the appliances. Nevertheless, our results outline that one must not expect longer overall treatment time, but more “considerable chair time” while treating patients with SHCNs.Our observation that patients with SHCNs commenced orthodontic treatment significantly earlier than patients with NSHCNs may be explained by the omnipresence of doctors for the SHCNs group and a more intensely observed maturation in these children (Figure 3).

### Pre-treatment PAR/AC

At admittance, differences in the PAR and AC scores between SHCNs and NSHCNs groups, to the disadvantage of the SHCNs group, could be detected. This observation could be explained by van der Linden et al., who identified 2 major factors influencing dentofacial morphology. They found genetic and environmental factors to determine dentofacial appearance [29]. Environmental factors such as generalized muscular hypotonia could often be found in patients with SHCNs [20]. However, literature on genetic factors is rare.

### Post-treatment PAR/AC

At the end of treatment, an inferior outcome for the SHCNs group compared to the NSHCNs group, as determined by the PAR and AC scores, was observed. These results may be attributed to the fact that parents and orthodontists of the SHCNs group focus more on the functional outcome and are willing to subordinate aesthetic effects. Further, less than adequate oral hygiene may force the orthodontist to reduce the treatment time in order to avoid dental damage, such as caries, periodontitis, or other [13]. Nevertheless, it must be mentioned that while statistically significant differences with respect to the AC scores did exist between both the patient groups, most of the patients with SHCNs achieved a post-treatment AC score of 4, i.e., they exhibited no further treatment need (Figure 6).

### Reduction in PAR/AC

Remarkably, no differences in the reduction of the PAR and AC scores could be found between the SHCNs and NSHCNs groups. Both groups showed a significant reduction in their PAR and AC scores. These findings raise the question of whether there is a genetic pre-disposition for orthodontic treatment outcome, as suggested by van der Linden et al. [29].

Our results provide limited insights into the treatment of a complex patient collective. However, they make it evident that there is a great need to promote orthodontic therapy options and make orthodontic treatment accessible to these children. Furthermore, they provide orthodontists with enough reason to not hesitate in treating members of this special needs group. Clearly, more research is needed to compare the effects of individual appliances used in orthodontics in order to identify the most suitable treatment modality for each individual CFA.

## Conclusions

The comparison of the overall treatment time and the number of appointments shows no significant differences between children/adolescents with SHCNs and NSHCNs.

Overall, more chair time is required in the patient group with SHCNs.

Differences exist in PAR and AC scores between children with SHCNs and NSHCNs at the time of admittance to orthodontic treatment, to the disadvantage of patients with SHCNs. Furthermore, an inferior treatment outcome rated by AC and PAR scores for children/adolescents with SHCNs is acknowledged.

### Ethical approval

The investigation was performed in compliance with the current revision of the Declaration of Helsinki, and with the International Conference for Harmonisation Good Clinical Practice (ICH-GCP) regulations and guidelines. Only data that was open to the authors for clinical practice was used. Referring to the Health Data Privacy Act of the County North Rhine-Westphalia § 6 sentence 2, the ethics committee of the Westphalian Wilhelms-University did not see reason for application (Letter dated Sept. 4th, 2013).

## Competing interests

The authors declare that they have no competing interests.

## Authors’ contributions

TS and AH conceptualized the paper. TS and MBL developed the study design. MBL collected the data, conducted the literature research, and authored the major portion of the manuscript. MBL performed the statistical analysis and contributed to the interpretation of the results. TS contributed towards statistical analysis and data handling, and reviewed the manuscript. DW reviewed the paper for content, including the final version of the manuscript. All authors have read and approved the final manuscript.

## Pre-publication history

The pre-publication history for this paper can be accessed here:

http://www.biomedcentral.com/1472-6831/14/67/prepub
